# Taxonomic and functional remodeling of the gut microbiota during aging and implications for microbiota-derived biomarkers

**DOI:** 10.1007/s11274-026-05017-6

**Published:** 2026-05-12

**Authors:** Burcu Tekin, Rafig Gurbanov

**Affiliations:** 1 Department of Biotechnology, Institute of Graduate Studies, Bilecik Şeyh Edebali University, Bilecik, Turkey; 2 Department of Bioengineering, Faculty of Engineering, Bilecik Şeyh Edebali University, Bilecik, Turkey; 3 Central Research Laboratory, Bilecik Şeyh Edebali University, Bilecik, Turkey

**Keywords:** Aging, Gut microbiota, Metagenomics, Microbial functional profiling, Microbiome, Microbiome-based biomarkers

## Abstract

The gut microbiota represents a complex microbial ecosystem that contributes to host metabolic regulation, immune homeostasis, and intestinal barrier function. Across the lifespan, gut microbial communities exhibit marked taxonomic and functional variation driven by environmental exposures, dietary patterns, medication use, and age-associated immune alterations. These differences are closely linked to chronic inflammatory states and immune dysregulation that accompany aging. This review synthesizes current evidence on age-associated differences in gut microbiota composition and functional capacity, with a focus on microbial traits and metabolic pathways relevant to host–microbe interactions. Pathological aging is frequently associated with reduced microbial diversity, loss of short-chain fatty acid–producing commensal bacteria, and enrichment of opportunistic or pro-inflammatory taxa. In contrast, healthy aging and longevity are commonly associated with more stable, resilient, and metabolically adaptable microbial communities. At the functional level, recurrent alterations in short-chain fatty acid biosynthesis, bile acid transformation, and tryptophan- and choline-related metabolic pathways define conserved features across aging-associated microbial profiles. Across neurodegenerative, metabolic, and cardiovascular conditions, overlapping taxonomic and functional patterns indicate shared microbiota-associated signatures linked to inflammatory states. Advances in metagenomic sequencing, functional annotation, and microbiome-focused biotechnological approaches now enable integrated analysis of microbial structure and metabolic potential. These developments provide a robust framework for identifying reproducible microbiome-based indicators relevant to aging-associated physiological changes and for translating microbiome research into biotechnology-driven applications.

## Introduction

The global population is undergoing an unprecedented demographic transition characterized by increased life expectancy and declining fertility rates. In 2020, approximately 727 million people were aged 65 years or older, and this number is projected to exceed 1.5 billion by 2050, increasing their global share from 9.3% to 16% (Nations 2020). By the 2070 s, individuals over 65 are expected to outnumber children under 18 (Economic, 2024). This demographic shift is accompanied by rising prevalence of chronic age-related diseases, including neurodegenerative, metabolic, and cardiovascular disorders, as well as sarcopenia and frailty (Niccoli and Partridge 2012; Organization 2015). Conventional clinical indicators often fail to detect early biological aging or subtle physiological decline, creating a demand for reliable, mechanism-based biomarkers capable of distinguishing healthy from pathological aging (López-Otín et al. 2013; Kennedy et al. 2014; Franceschi and Campisi 2014).

The gut microbiota has emerged as a particularly promising system in this context. This dense microbial ecosystem contributes to nutrient metabolism, immune regulation, and maintenance of epithelial homeostasis (Thursby and Juge 2017). Age-associated differences in long-term dietary patterns, medication exposure, and immune status are consistently accompanied by marked variation in microbial composition and function (O’Toole and Jeffery 2015; Biagi et al. 2010). These microbial differences parallel systemic processes of aging and are closely linked to its key immunological hallmarks, namely chronic low-grade inflammation (inflammaging) and immune senescence (immunosenescence) (Franceschi et al. 2017; Thevaranjan et al. 2017; Rosser and Mauri 2016).

Microbial profiles characterized by depletion of beneficial metabolically active commensals and enrichment of pro-inflammatory, oxygen-tolerant species are associated with increased intestinal permeability, enhanced endotoxin translocation, and activation of inflammatory signaling pathways (Buford 2017). This imbalance is thought to reinforce systemic inflammation and disrupts immune cell differentiation, thereby promoting a self-sustaining inflammatory milieu associated with tissue aging. Conversely, individuals exhibiting relatively balanced microbial networks often exhibit enhanced metabolic adaptability, efficient energy utilization, and reduced inflammatory tone, which are traits associated with extended health span and longevity (Odamaki et al. 2016; Biagi and Santoro 2022; Biagi et al. 2017).

Recent advances in high-throughput sequencing technologies, metagenomic functional annotation, and metabolomic profiling have substantially advanced the characterization of age-associated microbial communities and their metabolic outputs (Knight et al. 2018; Integrative 2014). These approaches enable integrative analysis of microbial structure, functional capacity, and host–microbe metabolic interactions, providing a foundation for microbiome-centered biotechnological applications and biomarker discovery in aging research (Chetty and Blekhman 2024; Sherwani et al. 2025; Duan et al. 2025).

This review synthesizes current evidence on the compositional and functional age-associated variation of the gut microbiota during aging, with particular emphasis on microbial mechanisms underlying inflammatory and immune alterations associated with age-related health deterioration. By integrating findings from experimental, clinical, and translational microbiome studies, it highlights conserved taxonomic and metabolic features that extend across multiple age-related disease contexts. Furthermore, this review discusses emerging methodological considerations and microbiome-focused analytical strategies relevant to the development of robust, reproducible, and biotechnology-driven microbiome-based indicators for aging research.

## Gut microbiota composition across the human lifespan

Human gut microbiota exhibits marked compositional variation across age groups, influenced by host physiology, diet, medication, and immune activity. These age-associated differences include both taxonomic and functional variation, which are closely linked to microbial resilience or dysbiosis in later-life cohorts. Distinct microbial configurations are observed among infancy, adulthood, senescence, and exceptional longevity cohorts, each reflecting a specific ecological equilibrium between host and microbes.

### Microbial diversity and richness in aging

Alpha diversity, integrating richness and evenness, is widely interpreted as a measure of microbial ecosystem stability (Lozupone et al. 2012). Commonly used metrics include the Shannon (Shannon 1948) and Simpson (Simpson 1949) indices, which quantify both the number of taxa and their relative distribution. These measures are sensitive to differences in community balance and are therefore useful for detecting ecological disruption or loss of microbial resilience. Their relationship with aging, however, remains inconsistent. In a large Italian cohort, Maffei et al. (2017) demonstrated that both observed and estimated richness were inversely correlated with biological age, measured by the frailty index (FI34), after adjusting for confounders (Maffei et al. 2017). Shannon diversity also declined with increasing frailty, whereas evenness indices were unaffected, suggesting that the observed changes may be driven by reductions in richness rather than shifts in dominance structure. However, the loss of rare taxa cannot be directly inferred from these indices alone and would require explicit richness-based measures, such as Chao1 or observed operational taxonomic units (OTUs) and amplicon sequence variants (ASVs), which are more sensitive to low-abundance taxa (Samuthpongtorn et al. 2022). In contrast, Zhang et al. (2020) reported no difference in Shannon or Simpson diversity between frail and non-frail elders, suggesting that frailty-diversity associations may depend on study design and cohort size (Zhang et al. 2020). These findings primarily reflect pathological aging contexts, particularly frailty-associated biological decline, whereas diversity patterns reported in healthy and exceptionally long-lived individuals appear partially distinct.

Large-scale cross-sectional analyses of healthy aging reveal divergent but generally more resilient diversity patterns. In 367 healthy Japanese individuals aged 0–104 years, Odamaki et al. (2016) documented a non-linear age-group distribution, with lower diversity in infancy, relative stabilization in adulthood, and higher diversity in centenarian cohorts (Odamaki et al. 2016). Similar non-linear age-group distributions were reported in Indian and Italian longevity cohorts (Biagi et al. 2016; Tuikhar et al. 2019) and implies that ecological diversification may accompany successful aging. Tuikhar et al. (2019) found significantly higher Chao1 richness in Indian centenarians than in younger adults, whereas Shannon diversity remained comparable, indicating richness gains without loss of community balance (Tuikhar et al. 2019). Inter-individual variation among centenarians was pronounced, emphasizing the influence of environment and lifestyle. Similarly, Kim et al. (2019) observed higher but non-significant diversity indices in independent centenarians compared with institutionalized peers in China, underscoring that living environment modulates microbial aging patterns (Kim et al. 2019).

Nevertheless, this relatively preserved diversity pattern in healthy longevity is not uniformly observed, and comparisons involving non-healthy or clinically compromised elderly populations continue to yield inconsistent results. Wu et al. (2019) found no significant differences in alpha diversity across age groups (Wu et al. 2019). Moreover, Singh et al. (2019) and Li et al. (2020) detected no diversity differences between healthy and non-healthy elders, while Zhang et al. (2021) found only a modest decline in unhealthy long-lived subjects (Singh et al. 2019; Li et al. 2020; Zhang et al. 2021). Together, these findings indicate that microbial diversity may increase, decrease, or remain stable depending on frailty, medication, and population context. Thus, alpha diversity per se is not a universal biomarker of healthy aging but rather reflects ecological stability conditional on host status.

Beta-diversity analyses offer clearer insight into age-group compositional differences. In the same Japanese cohort, Odamaki et al. (2016) showed that UniFrac distances significantly correlated with age (Odamaki et al. 2016). Biagi et al. (2016) demonstrated distinct clustering among centenarians, elderly, and adults, a pattern confirmed by Tuikhar et al. (2019) using Bray–Curtis dissimilarities and by Wu et al. (2019) in Chinese cohorts (Biagi et al. 2016; Tuikhar et al. 2019; Wu et al. 2019). Centenarians exhibited greater intra-group dispersion, indicating individualized community configurations likely shaped by lifelong environmental exposures. In contrast, Singh et al. (2019) observed minimal separation between healthy and non-healthy older adults aged 70–82 years, suggesting that major compositional divergence is more clearly detectable in extreme-age cohorts (Singh et al. 2019). These consistent beta-diversity differences support the idea that centenarian microbiota represents a distinct ecological state, potentially harboring biomarker taxa for resilience and longevity.

### Shifts in dominant microbial compositions

Age-associated gut microbiota differences are not limited to diversity indices but also include marked variation in the dominance and relative abundance of specific microbial groups. Across the lifespan, four phyla: Firmicutes, Bacteroidetes, Actinobacteria, and Proteobacteria dominate but vary in relative abundance (La-Ongkham et al. 2020; Odamaki et al. 2016). Infancy is characterized by Actinobacteria (mainly *Bifidobacterium*) and Proteobacteria (*Escherichia coli*), with low overall counts (Mariat et al. 2009). Adult cohorts are generally characterized by increased Firmicutes abundance, corresponding to a mature microbial profile enriched in Ruminococcaceae and Lachnospiraceae. In older individuals, Firmicutes are generally reported at lower relative abundance, whereas Bacteroidetes tend to be relatively increased, resulting in a reduced Firmicutes/Bacteroidetes (F/B) ratio. Across age groups, this ratio has been described as lowest in infancy, highest in adulthood, and relatively lower again in older populations (Mariat et al. 2009; Vaiserman et al. 2020). However, the F/B ratio is increasingly regarded as an overly simplified metric, as it fails to capture the substantial intra-phylum functional heterogeneity revealed by modern metagenomic analyses (Nez-Castro et al. 2026; Magne et al. 2020). Proteobacteria is reported at markedly higher relative abundance in cohorts above 70 years, while Synergistetes and Christensenellaceae often expand in centenarians (Odamaki et al. 2016; Biagi et al. 2016; Wang et al. 2019).

Within Firmicutes, Lachnospiraceae and Ruminococcaceae dominate adult microbiota but are reported at lower relative abundance in extreme aging cohorts, whereas subdominant families such as Christensenellaceae, Synergistaceae, and Mogibacteriaceae are more prominently represented in these long-lived groups (Biagi et al. 2016). Similarly, Bacteroidaceae, Porphyromonadaceae, and Prevotellaceae show relatively higher abundance in elderly and longevity-associated cohorts compared with younger adults (Wang et al. 2019; La-Ongkham et al. 2020). Bifidobacteriaceae remain higher in adults than in elderly individuals (La-Ongkham et al. 2020).

Genus-level analyses reveal marked compositional differences with functional implications. Beneficial short-chain fatty acid (SCFA) producers such as *Faecalibacterium*, *Roseburia*, and *Coprococcus* are consistently reported at lower abundance in older cohorts (Wang et al. 2018; Biagi et al. 2016). Meanwhile, *Bacteroides*, *Parabacteroides*, *Oscillospira*, *Odoribacter*, *Desulfovibrio*, *Akkermansia*, and *Bilophila* are relatively enriched in older cohorts (Biagi et al. 2016; Xu et al. 2019; La-Ongkham et al. 2020). Notably, Wilmanski et al. (2021) showed that healthy aging is associated with increased microbiome uniqueness and a decline in core taxa, particularly *Bacteroides*, whereas the persistence of high *Bacteroides* abundance in advanced age is linked to reduced survival (Wilmanski et al. 2021). *Escherichia–Shigella* enrichment is frequently observed in late-life cohorts, reflecting opportunistic expansion under immunosenescence (Wang et al. 2018). *Lactobacillus* shows non-linear abundance differences across age cohorts, with intermediate enrichment reported in some studies (Xu et al. 2019). Co-abundance group analyses indicate age-group-specific dominance patterns: *Bifidobacterium* in infancy, Lachnospiraceae in adulthood, and *Bacteroides*/Clostridiaceae in old age (Odamaki et al. 2016).

At species resolution, adults display *Dorea longicatena* and *Clostridium bartlettii*, whereas elderly groups harbor *Bacteroides uniformis*, *Streptococcus salivarius*, and *Parabacteroides distasonis* (La-Ongkham et al. 2020). Certain species, including *Bacteroides fragilis*, *Parabacteroides merdae*, *Ruminococcus gnavus*, and *Coprococcus* spp., have been associated with longevity in specific cohorts, while *Bacteroides vulgatus* and some *Ruminococcus* and *Clostridium* taxa decline (Wang et al. 2019). In contrast to the general age group–associated patterns described above, the positive association of *Coprococcus* with longevity may reflect features specific to extreme longevity, as well as underlying species- or strain-level heterogeneity within the genus. Core species such as *Faecalibacterium prausnitzii*, *Blautia wexlerae*, and *Ruminococcus obeum* are consistently detected across multiple age cohorts, suggesting a relatively conserved microbial backbone despite age-associated compositional differences (La-Ongkham et al. 2020).

Cross-sectional comparisons indicate broad compositional differences between infant, adult, elderly, and longevity-associated microbiota states, characterized by variation in dominant phyla, reduced abundance of selected core Firmicutes, increased Proteobacteria representation, and enrichment of low-abundance functional taxa in advanced-age cohorts (Mariat et al. 2009; Biagi et al. 2016; Odamaki et al. 2016; La-Ongkham et al. 2020; Vaiserman et al. 2020). Overall, these age-group differences provide a robust set of microbial indicators reflecting broad host-microbe ecological variation across the lifespan and offer potential references for identifying age-associated gut microbial patterns and supporting healthy longevity.

### Functional microbial signatures of age and longevity

Taxonomic differences only partially explain aging-related physiology, whereas functional capacity provides mechanistic clarity. Microbial metabolites such as SCFAs, aromatic amino-acid derivatives, methylamines, and bile acids (BAs) mediate host–microbe crosstalk and act as candidate biomarkers of healthy or pathological aging.

Butyrate, propionate, and acetate, generated through fermentation of dietary polysaccharides and amino acids, sustain colonic energy metabolism and immune regulation (Hamer et al. 2008; Langley et al. 2008; Zhou et al. 2021). Butyrate serves as the principal energy source for colonocytes (Hamer et al. 2008) and exerts immunomodulatory effects through histone deacetylase inhibition (Langley et al. 2008). Its biosynthesis proceeds mainly through the butyryl-CoA/acetate CoA-transferase and phosphotransbutyrylase–butyrate-kinase pathways (Louis and Flint 2009). Elderly individuals show reduced abundance of the butyryl-CoA/acetate CoA-transferase gene, implying diminished saccharolytic capacity (Hippe et al. 2011). Reduced abundance of canonical butyrate producers (*Faecalibacterium*, *Coprococcus*, *Roseburia*) is a recurrent finding across older cohorts (Biagi et al. 2016). Nonetheless, centenarians show enrichment of alternative amino-acid-fermenting taxa (*Alistipes*, *Parabacteroides*, *Odoribacter*, *Porphyromonas*) that maintain SCFA pools (Tuikhar et al. 2019). Metagenomic data reveal enhanced glycolysis, pentose-phosphate, and TCA cycle genes in centenarians, suggesting preserved energy metabolism (Wu et al. 2019). Although genes for amino-acid biosynthesis decline, proteolytic fermentation pathways expand (Rampelli et al. 2013), indicating greater reliance on amino-acid substrates relative to carbohydrate fermentation. This metabolic flexibility may contribute to longevity by sustaining epithelial nutrition and dampening inflammation despite taxonomic turnover.

Beyond SCFAs, aromatic amino acid metabolism yields metabolites with potential as aging biomarkers. Altered phenylalanine and tyrosine metabolism correlates with increased urinary phenylacetylglutamine (PAGln) and p-cresol sulfate (PCS) in centenarians (Rampelli et al. 2013). Higher levels of PAGln, PCS, and 2-hydroxybenzoate were confirmed in centenarian urine, with PAGln positively correlating with Proteobacteria species such as *E. coli* and *Pseudomonas* (Collino et al. 2013). These observations have been interpreted as reflecting age-associated shifts in host–microbiome metabolic interactions, suggesting coordinated changes in amino acid metabolism and gut microbiota function. However, the interpretation of their accumulation in centenarians remains complex and may reflect multiple, not mutually exclusive mechanisms, including both microbial and host-related factors.

Trimethylamine (TMA), a product of microbial metabolism of choline, carnitine, and related dietary amines, is oxidized in the liver to trimethylamine N-oxide (TMAO). Elevated plasma TMAO levels are generally reported in older adults and are associated with cardiovascular risk, including arterial stiffness and higher mortality in peripheral artery disease (Senthong et al. 2016; Brunt et al. 2020; Mohsenzadeh et al. 2025). Gut microbiota composition influences this pathway, with elderly individuals showing higher fecal TMA and choline levels when their microbiota displays an “elderly-type” configuration (Yoshimoto et al. 2021).

Tryptophan catabolites such as indole-3-acetate and kynurenic acid activate the aryl hydrocarbon receptor (AhR), strengthening epithelial barriers and anti-inflammatory signaling (Tang et al. 2024). Proteomic comparisons across age cohorts indicate lower TnaA/TrpB enzyme abundance in older individuals than in younger groups (Ruiz-Ruiz et al. 2020). Reduced indole production is linked to increased intestinal permeability and neuroinflammation (Santoro et al. 2018). Healthy centenarians display elevated serum 5-methoxyindoleacetic acid (5-MIAA), kynurenic acid, and indole-3-acetate levels correlated with Christensenellaceae R-7 group abundance (Qiu et al. 2025), suggesting preservation of the tryptophan–AhR axis as a hallmark of longevity.

The microbial conversion of primary BAs into secondary bile acids (SBAs) is a major microbiota-driven metabolic process influencing host physiology. Primary BAs, synthesized from cholesterol and conjugated in the liver, are deconjugated and 7α-dehydroxylated in the intestine by bile salt hydrolases (BSHs) and hydroxysteroid dehydrogenases (HSDHs) from *Clostridium*, *Bacteroides*, *Eubacterium*, *Lactobacillus*, and *Escherichia* (Jin et al. 2024; Chen et al. 2019). The resulting SBAs, mainly deoxycholic acid (DCA) and lithocholic acid (LCA), act as potent agonists of farnesoid X receptor (FXR) and Takeda G protein-coupled receptor 5 (TGR5), regulating glucose and lipid metabolism, energy balance, and inflammation (Wang and Zhao 2018; Lu et al. 2024; Perino et al. 2021). In germ-free models, accumulation of FXR-antagonistic BAs such as tauro-β-muricholic acid (TβMCA) demonstrates the microbiome’s major role in shaping BAs composition (Sayin et al. 2013). Thus, BA transformation and microbiota form a reciprocal regulatory system involved in metabolic homeostasis. Notably, centenarian microbiomes have been reported to be enriched in bacteria capable of producing structurally distinct SBAs, including isoallolithocholic acid, via specific enzymatic pathways, with potential antimicrobial activity against Gram-positive pathogens, suggesting a role in maintaining intestinal homeostasis (Sato et al. 2021).

Collectively, available cohort comparisons suggest that healthy and extreme aging are characterized less by taxonomic constancy than by preservation of key metabolic pathways. SCFA synthesis, aromatic-amino-acid catabolism, TMA/TMAO turnover, tryptophan–AhR signaling, and bile-acid transformations constitute functional hallmarks of microbial aging. Their quantitative balance may provide more informative indicators of biological age than diversity metrics alone, offering measurable biomarkers for distinguishing healthy from pathological aging states in later life. Nevertheless, it should be recognized that while several of these functional signatures are supported by direct metabolomic, metagenomic, or experimental evidence, part of the current interpretation in aging microbiome research still relies on predicted metabolic capacity inferred from taxonomic profiles.

## Gut microbiota as a central regulator of inflammaging and immunosenescence

Aging involves two intertwined immunological phenomena: inflammaging, a chronic low-grade inflammatory state, and immunosenescence, the gradual deterioration of immune competence. These processes, while predisposing to age-related disease, also represent adaptive remodeling to lifelong antigenic and metabolic stress (Santoro et al., 2020). Centenarians exemplify successful aging by achieving a balanced pro- and anti-inflammatory equilibrium shaped by their “immunobiography” (Franceschi et al. 2017). The gut microbiota (an evolutionarily co-adapted ecosystem) acts as a central regulator of these immunological alterations, linking environmental exposures to the host immune system, while host mucosal immune mechanisms, including secretory IgA, antimicrobial peptides, and epithelial barrier components, reciprocally regulate microbial composition and spatial organization (Bischoff 2016; Dominguez-Bello et al. 2019; Belkaid and Hand 2014).

Age-associated dysbiosis is commonly characterized by several recurrent patterns: (i) depletion of anti-inflammatory, butyrate-producing taxa such as *Faecalibacterium* and *Roseburia*; (ii) context-dependent alterations in microbial diversity, which may include decreases, stability, or increases depending on host and environmental factors; and (iii) expansion of pro-inflammatory pathobionts from Enterobacteriaceae, Streptococcaceae, and Staphylococcaceae (Biagi et al. 2016; Santoro et al. 2018; Toward et al. 2012). Declining SCFA production (especially butyrate) plays a pivotal mechanistic role, as SCFAs sustain gut barrier integrity and immune homeostasis. Butyrate suppresses NF-κB signaling, reinforces tight-junction proteins, and promotes regulatory T- and B-cell differentiation (Koh et al. 2016; Rosser et al. 2020; Conway and Duggal 2021). Reduced SCFA availability is therefore thought to amplify inflammatory signaling loops (Medina et al. 2019). Simultaneously, enrichment of Proteobacteria, notably *Escherichia*–*Shigella*, elevates luminal lipopolysaccharide (LPS), a potent immune activator (Shin et al. 2015).

Barrier dysfunction represents one of the major downstream consequences associated with these microbial alterations. Older cohorts are frequently reported to exhibit a thinner mucus layer and reduced expression of tight-junction proteins such as zonulin and occludin, producing a “leaky gut” (Elderman et al. 2017; Tran and Greenwood-Van Meerveld 2013; Wilson et al. 2018). Translocated microbial products (LPS and other microbe-associated molecular patterns (MAMPs)) engage Toll-like receptors (TLRs) and related pattern-recognition receptors (PRRs) on immune cells, activating NF-κB and driving persistent secretion of IL-6, TNF-α, and IL-8 (Dominguez-Bello et al. 2019; Conway and Duggal 2021). Chronic, low-grade endotoxemia is therefore considered a major molecular contributor to inflammaging (Thevaranjan et al. 2017).

Specific taxa correlate reproducibly with systemic inflammatory markers, supporting their relevance as candidate microbial biomarkers. *Alistipes*, *Parabacteroides*, *Escherichia*, and *Shigella* abundance positively correlates with plasma IL-8 and CRP (Claesson et al. 2012), while increased Proteobacteria parallels higher TNF-α and IL-6. The roles of *Alistipes* and *Parabacteroides* appear to vary across host and environmental contexts, as these genera have also been associated with beneficial metabolic functions in centenarians, potentially reflecting species- or strain-level heterogeneity. Butyrate producers such as *Faecalibacterium* and *Anaerostipes* show inverse correlations (Biagi et al. 2010; Wu et al. 2022a). Circulating LPS and inflammatory cytokines themselves (IL-6, TNF-α, IL-8) constitute validated biomarkers of inflammaging (Thevaranjan et al. 2017; DeJong et al. 2020). Together, variation in microbial diversity and the balance between pro- and anti-inflammatory taxa may contribute to a composite microbial signature associated with age-related inflammation and immune decline.

### Evidence from animal and human studies

Experimental models provide substantial mechanistic evidence that microbiota composition drives inflammaging. Germ-free mice are largely protected from age-related inflammation but develop elevated cytokines once colonized with aged microbiota, supporting a direct microbial contribution (Thevaranjan et al. 2017). Fecal transfer from aged donors to young germ-free recipients induces TNF-α upregulation and TLR activation, features associated with immunosenescence (Fransen et al. 2017). Transplantation of aged microbiota also aggravates neuroinflammation and worsens stroke outcomes (Spychala et al. 2018). Across models, *Parabacteroides* and *Mucispirillum* correlate positively with inflammatory markers, whereas *Akkermansia* and *Lactobacillus* exhibit anti-inflammatory associations (Conley et al. 2016).

Human cohort studies provide translational support for these observations. Dysbiotic microbiota enriched in Proteobacteria and depleted in SCFA-producing Firmicutes consistently accompany aging (Biagi et al. 2010; Wu et al. 2022a). Proteobacterial expansion aligns with higher plasma TNF-α and IL-8, while *Faecalibacterium* and *Anaerostipes* abundance inversely tracks these cytokines (Wu et al. 2022a). Even in successful aging, centenarians display distinct microbial–cytokine pairings, in which certain Proteobacteria correlate with IL-6 and IL-8 elevations, reflecting adaptive but persistent immune activation (Biagi et al. 2010). The recurrence of these patterns across species supports the consistency of microbial–inflammatory coupling. Taxa inducing inflammation in murine models show partial overlap with genera linked to systemic cytokines in humans (Conley et al. 2016; Wu et al. 2022a). However, these comparisons should be interpreted with caution due to inherent differences between murine and human gut microbiota, particularly as certain taxa such as *Mucispirillum* are common in rodents but rare or absent in humans (Herp et al. 2021). Similarly, anti-inflammatory taxa identified in animal models are often associated with reduced inflammation in human cohorts, suggesting partially conserved anti-inflammatory associations.

The clinical relevance is underscored by longitudinal studies showing that elevated IL-6 and TNF-α, key inflammatory correlates of dysbiosis, predict morbidity and mortality (Brüünsgaard and Pedersen 2003; DeJong et al. 2020).

Collectively, convergent evidence from animal and human research supports a mechanistic link between microbial ecology, gut permeability, and systemic inflammation. Within this framework, specific bacterial consortia, their metabolites, and corresponding cytokine profiles emerge as candidate biomarkers for evaluating immunological aging and resilience.

## Gut microbiota in age-related diseases

**Fig. 1:**
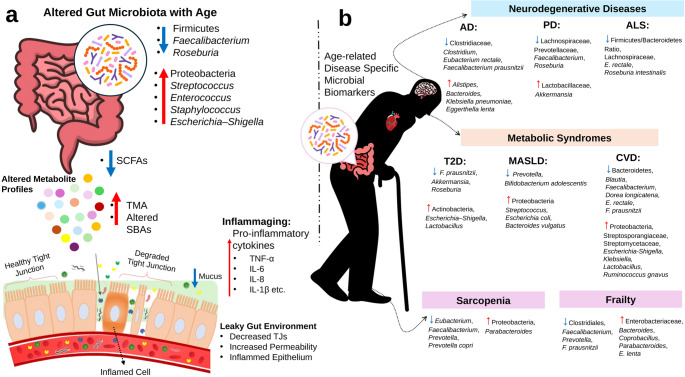
Age-associated gut microbiota alterations and disease-specific microbial signatures. **a** Overview of microbial compositional and functional changes occurring during normal aging. **b** Representative microbial signatures in major age-related diseases SCFA, short-chain fatty acid; TMA, trimethylamine; SBA, secondary bile acid; AD, Alzheimer’s disease; PD, Parkinson’s disease; ALS, amyotrophic lateral sclerosis; T2D, type 2 diabetes; MASLD, metabolic dysfunction-associated steatotic liver disease; CVD, cardiovascular disease

The preceding sections highlight the contrast between the microbial configuration associated with healthy aging and the dysbiotic, pro-inflammatory milieu characteristic of inflammaging. This divergence leads to a pivotal question: how are these broad microbial patterns reflected in specific disease-associated signatures? This section synthesizes evidence linking specific gut microbiota alterations and their metabolic consequences to major age-related diseases, including neurodegenerative, metabolic, and musculoskeletal conditions. An overview of these age-associated microbial differences and their disease-specific associations is illustrated in Fig. 1. These patterns also represent disease-specific associations built upon the shared dysbiotic core discussed in Section 5.

### Neurodegenerative diseases

Neurodegenerative disorders such as Alzheimer’s disease (AD), Parkinson’s disease (PD), and amyotrophic lateral sclerosis (ALS) exemplify how age-associated microbial differences intersect with systemic inflammation, metabolic remodeling, and neuroimmune dysfunction. The gut–brain axis serves as the physiological interface through which these microbial perturbations modulate neurodegeneration via immune, endocrine, and neural pathways (König et al. 2016; Zhan et al. 2018). Age-related dysbiotic microbial profiles are frequently associated with impaired intestinal permeability, increased translocation of LPS, and activation of TLR pathways, triggering NF-κB–dependent cytokine cascades (Zhao et al. 2015). Concurrently, reduced abundance of SCFA producers such as *Faecalibacterium* and *Roseburia* reduces butyrate availability, a key anti-inflammatory metabolite that normally reinforces barrier integrity and modulates immune tolerance (Koh et al. 2016; Rosser et al. 2020). In contrast, expansion of Enterobacteriaceae and other Proteobacteria elevates LPS burden and systemic endotoxemia, perpetuating a pro-inflammatory milieu.

Beyond inflammation, microbial metabolites directly influence neuronal activity. Gut-derived neurotransmitters such as serotonin, γ-aminobutyric acid (GABA), dopamine, and histamine are produced by specific taxa including *Lactobacillus*, *Bifidobacterium*, *Streptococcus*, and *Escherichia* (Strandwitz 2018; Kyei-Baffour et al. 2025). Age-associated dysbiosis may alter their production, reducing the availability of neuroactive compounds critical for synaptic plasticity and cognitive function (Askarova et al. 2020). Moreover, bacterial amyloids such as curli fibers produced by Proteobacteria can cross-seed with β-amyloid (Aβ) aggregates and activate TLR2 signaling, amplifying glial inflammation (Rapsinski et al. 2015; Zhao and Lukiw 2018). Together, these mechanisms suggest that microbiota-dependent systemic and local neuroinflammation represents a common mechanistic axis potentially underlying late-life neurodegenerative disease.

#### Alzheimer’s disease (AD)

Alzheimer’s disease (AD), the most prevalent cause of dementia, is pathologically defined by extracellular Aβ plaques and intracellular hyperphosphorylated tau tangles, leading to neuroinflammation, synaptic dysfunction, and neuronal loss (Hu et al. 2016; Kowalski and Mulak 2019; Jouanne et al. 2017). Growing evidence implicates gut microbiota as a key regulator of these neurodegenerative processes via immune, metabolic, and neurochemical pathways.

Gut dysbiosis is consistently reported in AD, though the specific taxa differ among studies. Reductions in butyrate-producing genera such as *F. prausnitzii*, *Roseburia*, and *Eubacterium*, alongside enrichment of pro-inflammatory taxa including *Bacteroides*, *Alistipes*, and *Odoribacter*, have been observed (Haran et al. 2019; Vogt et al. 2017). These microbial differences correspond with diminished SCFA production and enhanced systemic inflammation. Some cohorts, however, reveal contrasting trends such as decreased Bacteroidetes or elevated Actinobacteria likely reflecting dietary and geographic variability (Zhuang et al. 2018). Despite inconsistencies, most findings converge on reduced microbial diversity and loss of anti-inflammatory metabolic capacity.

Comparisons across distinct disease stages suggest that microbial community differences are detectable early in the clinical course of AD. In individuals with mild cognitive impairment, taxa such as *Faecalibacterium*, *Dorea*, *Oscillibacter*, and *Coprococcus* show altered abundance, indicating that microbial differences are detectable even at the mild cognitive impairment stage (Ferreiro et al. 2023). In clinically advanced AD cohorts, enrichment of *Alistipes*, *Bacteroides*, and *Prevotella* has been associated with intensified inflammatory and neurochemical imbalance (Jia et al. 2025).

Microbial metabolites also contribute mechanistically to AD pathology. Reduced fecal SCFA levels, particularly butyrate and propionate, have been associated with increased Aβ deposition and impaired gut barrier integrity (Zhang et al. 2017; Zheng et al. 2019). Consistent with these observations, SCFAs have been reported to inhibit Aβ aggregation and exert neuroprotective effects (Ho et al. 2018). As noted above, bacterial curli fibers may further facilitate Aβ aggregation.

Collectively, AD is consistently associated with depletion of anti-inflammatory SCFA-producing taxa, enrichment of inflammatory LPS-associated genera, and early microbial alterations detectable before severe cognitive decline. These recurrent signatures emphasize the potential of microbiota-based interventions and early biomarker discovery in AD (Vogt et al. 2017; Haran et al. 2019; Ferreiro et al. 2023; Jia et al. 2025).

#### Parkinson’s disease (PD)

Parkinson’s disease (PD) is one of the strongest clinical contexts supporting a gut-to-brain propagation model of neurodegeneration. Histopathological data suggest that α-synuclein aggregation may initially involve the enteric nervous system (ENS) before reaching the brain (Campos-Acuña et al. 2019). Aging-related intestinal barrier impairment is thought to facilitate microbial translocation and endotoxemia, thereby promoting local immune activation and systemic oxidative stress (Forsyth et al. 2011; Caputi and Giron 2018). Elevated circulating LPS disrupts blood–brain barrier integrity, while reduced mucin synthesis and SCFA production weaken epithelial defense.

Several studies have described characteristic alterations in gut microbiota composition in PD. Scheperjans et al. (2015) reported increased Enterobacteriaceae correlating with motor severity and reduced Prevotellaceae, which are involved in mucin synthesis, SCFA production, and vitamin biosynthesis (Scheperjans et al. 2015). Their depletion has been associated with increased intestinal permeability and exposure to microbial metabolites (De Vadder et al. 2014; Forsyth et al. 2011). Reduced Prevotellaceae and elevated Lactobacillaceae have also been linked to lower ghrelin levels, a hormone affecting dopaminergic function (Andrews et al. 2009; Unger et al. 2011).

Further studies found enrichment of *Ralstonia* and loss of butyrate-producing *Blautia*, *Coprococcus*, and *Roseburia* (Keshavarzian et al. 2015; Aho et al. 2019). Hopfner et al. (2017) observed increased Lactobacillaceae, Enterococcaceae, and Barnesiellaceae (Hopfner et al. 2017). Meta-analyses revealed consistent patterns across cohorts: higher *Akkermansia* and Lactobacillaceae, and lower *Roseburia*, *Faecalibacterium*, and Lachnospiraceae (Nishiwaki et al. 2020; Cirstea et al. 2020; Wallen et al. 2020; Barichella et al. 2019). Notably, although *Akkermansia* is generally considered beneficial, its increased abundance in PD is widely hypothesized to represent a secondary consequence of disease-associated gastrointestinal dysfunction, particularly severe constipation and delayed intestinal transit. As a mucin-degrading bacterium, *Akkermansia muciniphila* may expand under conditions of altered mucus turnover and reduced fiber availability, and this shift may further weaken barrier integrity and enhance intestinal permeability (Kleine Bardenhorst et al. 2023; Fang et al. 2021). However, further longitudinal and mechanistic studies are required to determine whether these changes are causative or reflect secondary adaptations to disease-associated gastrointestinal dysfunction.

Overall, PD is associated with a microbial profile marked by reduced SCFA-producing taxa and enrichment of pro-inflammatory bacteria, consistent with intestinal and metabolic imbalance. However, interpretation of these signatures is complicated by medication-related confounding. In particular, gut bacteria can metabolize L-3,4-dihydroxyphenylalanine (levodopa), the primary dopaminergic precursor used in PD treatment, thereby reducing its bioavailability and influencing therapeutic response. However, findings regarding the effects of levodopa on gut microbiota composition remain heterogeneous across studies, likely due to differences in cohort composition, disease stage, and treatment regimens (Menozzi and Schapira 2024).

#### Amyotrophic lateral sclerosis (ALS)

Amyotrophic lateral sclerosis (ALS) is a progressive neurodegenerative disorder characterized by the degeneration of upper and lower motor neurons, resulting in paralysis and respiratory failure. Neuroinflammation and microglial activation are major pathological features, suggesting an immunological component in disease pathophysiology (Zhu et al. 2021). Growing evidence indicates that gut microbiota alterations may interact with these processes by influencing intestinal barrier integrity and neuroimmune signaling along the gut–brain axis.

Initial clinical observations suggest that gut microbial differences are already detectable in ALS patients. Clinical studies consistently report reduced microbial diversity together with depletion of butyrate-producing Firmicutes in ALS. Reduced F/B ratio and lower abundance of taxa such as *Anaerostipes*, *Oscillibacter*, *Lachnospiraceae*, *Eubacterium rectale*, and *Roseburia intestinalis* have been documented, supporting impaired SCFA biosynthesis and disrupted mucosal homeostasis (Spielman et al. 2018; Pellegrini et al. 2018; Nicholson et al. 2021). Larger cohort analyses similarly reported increased Bacteroidetes and reduced Firmicutes, reinforcing the consistency of these dysbiotic patterns (Zeng et al. 2020). Although these phylum-level alterations may not be entirely disease-specific, they remain broadly consistent with reduced microbial diversity and impaired SCFA biosynthesis.

Beyond observational data, mechanistic evidence from animal models supports a causal role of the gut microbiota in ALS. In microbiota-manipulated SOD1-Tg mice, depletion of the microbiome exacerbated disease severity, whereas supplementation with specific taxa such as *A. muciniphila* ameliorated motor symptoms and disease progression, partly through microbiota-derived metabolites including nicotinamide (Blacher et al. 2019). These findings suggest that microbiota–host interactions may contribute to neurodegenerative processes. However, given that human data remain largely observational and heterogeneous, further longitudinal and mechanistic studies are required to clarify the causal relevance of these associations.

### Metabolic syndromes

Metabolic syndromes such as type 2 diabetes (T2D), metabolic dysfunction-associated steatotic liver disease (MASLD), and cardiovascular disease (CVD) are highly prevalent in older adults and represent key components of age-related metabolic decline. As discussed earlier, aging-associated gut microbiota differences commonly include reduced diversity, depletion of beneficial taxa, and enrichment of pro-inflammatory species, patterns that are closely associated with metabolic dysregulation. In older cohorts, gut dysbiosis has been linked to insulin resistance, impaired lipid metabolism, and persistent low-grade inflammation. This section examines the gut microbial signatures and functional alterations associated with T2D, MASLD, and CVD in the context of aging, emphasizing shared and disease-specific microbial biomarkers.

#### Type 2 diabetes (T2D)

Type 2 diabetes (T2D) is a metabolic disorder strongly associated with older age and highly prevalent in elderly populations (Ling et al. 2022). Beyond glucose dysregulation, it increases the risk of metabolic syndrome, CVD, and cerebrovascular complications. Growing evidence suggests that gut microbiota alterations may act as microbial indicators of systemic inflammation, metabolic imbalance, and insulin resistance (Iatcu et al. 2021; Ruuskanen et al. 2022; Chong et al. 2025).

Metagenomic and 16S rRNA studies consistently report reduced microbial diversity together with depletion of butyrate-producing commensals and enrichment of pro-inflammatory or oxidative stress–associated taxa in T2D. Specifically, reduced abundance of *Roseburia*, *Faecalibacterium*, *Akkermansia*, *Bifidobacterium*, *Bacteroides*, *Prevotella*, *Clostridium*, and Peptostreptococcaceae, alongside enrichment of *Lactobacillus*, *Escherichia–Shigella*, *Enterococcus*, *Subdoligranulum*, *Fusobacterium*, *Collinsella*, *Actinobacteria*, Coriobacteriaceae, and *Ruminococcus*, has been repeatedly documented, collectively supporting a reproducible inflammatory-microbial signature linked to impaired SCFA biosynthesis, increased endotoxin burden, and altered glucose metabolism (Qin et al. 2012; Afolayan et al. 2020; Chong et al. 2025). Older age appears to influence the strength and consistency of these associations. Enqi et al. (2019) further reported that elevated glucose levels correlated with higher *Actinobacteria*, *Bifidobacterium*, and *Lactobacillus* in older adults, although some taxa, particularly *Bifidobacterium*, remain inconsistently associated across cohorts, likely due to differences in age structure, medication use, comorbidities, and analytical methodology (Enqi et al. 2019). Accordingly, while several candidate microbial biomarkers have emerged, more age-stratified and longitudinal studies are needed to define robust microbiota-based indicators for T2D in older adults.

#### Metabolic dysfunction-associated steatotic liver disease (MASLD)

Metabolic dysfunction-associated steatotic liver disease (MASLD), formerly referred to as non-alcoholic fatty liver disease (NAFLD), is the most common chronic liver disorder worldwide, affecting about one-quarter of adults and increasing the risk of CVD and hepatocellular carcinoma (Zeng et al. 2024). It is a multifactorial condition shaped by genetic, metabolic, and inflammatory factors, with gut microbiota dysbiosis and gut–liver axis disruption playing key roles (Ling et al. 2022). Mechanistically, gut microbiota have been implicated in MASLD through increased intestinal permeability and LPS translocation, altered BA metabolism, reduced choline availability, and enhanced intestinal ethanol production, factors closely associated with hepatic inflammation and steatosis (Safari and Gérard 2019).

Clinical and metagenomic studies consistently link gut microbial differences to MASLD severity. Biopsy-confirmed MASLD has been associated with enrichment of *Bacteroides* and *Ruminococcus* and depletion of *Prevotella*, with *Bacteroides* dominance correlating with steatohepatitis, fibrosis, and altered carbohydrate, lipid, and amino acid metabolism (Boursier et al. 2016). Additional studies reported increased *Escherichia*, *Lactobacillus*, *Streptococcus*, Proteobacteria, and *E. coli* accompanied by elevated intestinal TNF-α and IL-6, together with depletion of butyrate-producing taxa such as *E. rectale* and *R. obeum*, supporting a dysbiotic profile linked to intestinal inflammation, oxidative stress, and endotoxemia (Jiang et al. 2015; Loomba et al. 2017). Recent large-scale analyses further identified *Fusobacteria* and *Veillonellaceae* as risk-associated taxa, whereas *Rikenellaceae*, *Barnesiellaceae*, and *Bifidobacterium adolescentis* showed protective associations (Zeng et al. 2024). In addition, sex-specific differences have been reported, with men showing reduced α-diversity and enrichment of *Dialister*, *Streptococcus*, and *Bifidobacterium*, whereas women exhibit relatively higher diversity and increased *Butyricimona*s abundance, a pattern that may be related to hormonal modulation of BA composition and microbial selection pressure (Shi et al. 2021).

Current evidence supports a MASLD-associated gut microbial configuration linked to endotoxemia, altered BA metabolism, and hepatic inflammatory-fibrotic burden. These recurrent associations highlight the gut–liver axis as a promising source of candidate microbial biomarkers, although longitudinal and age-focused studies are still required to clarify their causal relevance.

#### Cardiovascular diseases (CVD)

Cardiovascular diseases (CVD) are the leading cause of morbidity and mortality in older adults. Beyond conventional risk factors, growing evidence associates gut microbiota dysbiosis with cardiovascular pathology through altered production of TMAO, BAs, and SCFAs, together with systemic inflammation, oxidative stress, and metabolic dysfunction (Matacchione et al. 2024).

In elderly heart failure (HF) cohorts, studies consistently report lower microbial diversity together with depletion of Coriobacteriaceae, Erysipelotrichaceae, Ruminococcaceae, *Faecalibacterium*, *F. prausnitzii*, and *Clostridium clostridioforme*, whereas *Escherichia–Shigella*, *Enterococcus*, *Klebsiella*, *Lactobacillus salivarius*, *R. gnavus*, *Veillonella*, and other Proteobacteria are recurrently enriched (Luedde et al. 2017; Sun et al. 2022; Kamo et al. 2017; Cui et al. 2018). These taxonomic changes parallel elevated microbial genes involved in TMAO synthesis, reduced butyrate-associated pathways, and diminished anti-inflammatory potential.

Integrative microbiome–metabolomic analyses further link enrichment of *R. gnavus*, *Escherichia–Shigella*, *Klebsiella*, *Haemophilus*, and *Lactobacillus* with elevated inflammatory cytokines (IL-6, TNF-α) and adverse circulating metabolites including bilirubin, ethyl salicylate, and PAGln. In addition, *Klebsiella* and *Lactobacillus* appear more prominent in severe HF, whereas *Bifidobacterium* is relatively enriched in moderate disease stages (Wang et al. 2021b; Zhang et al. 2023b).

Similar dysbiotic signatures extend to other CVD subtypes. Elderly coronary disease cohorts show Proteobacteria enrichment, including *Klebsiella pneumoniae*, *Desulfovibrio desulfuricans*, and *Burkholderia*, together with higher fecal TMA levels (He et al. 2024). In hypertension, reduced alpha diversity and *Escherichia–Shigella* enrichment contrast with beneficial associations of SCFA-producing Lachnospiraceae and *Ruminococcus* UCG-002 (Valdez Palomares et al. 2025). In cerebrovascular disease, reduced *Blautia obeum* and increased *Streptococcus infantis* and *Prevotella copri* have been linked to enhanced LPS and flagellar synthesis pathways (Huang et al. 2019).

CVD in older adults is consistently associated with a gut microbial-metabolic profile marked by impaired butyrate support, increased endotoxemia, and enhanced generation of atherogenic metabolites such as TMAO and PAGln. These recurrent associations highlight the gut microbiota as a promising mechanistic and prognostic biomarker source in cardiovascular aging.

### Sarcopenia and frailty

Sarcopenia is a progressive, age-related loss of skeletal muscle mass and function that increases the risk of falls, frailty, and mortality (Cruz-Jentoft et al. 2010; Dennison et al. 2017). Its pathogenesis involves chronic inflammation, oxidative stress, anabolic resistance, and reduced physical activity (Wilson et al. 2017). Increasing evidence links gut microbiota dysbiosis to sarcopenia through immune–metabolic disturbances, reduced SCFA production, and systemic inflammation.

Multiple studies have shown that gut microbiota diversity and composition differ between sarcopenic and healthy older adults, although alpha-diversity findings remain inconsistent across cohorts. Several cohorts reported reduced α-diversity indices (Chao1, Shannon, Simpson) in sarcopenia (Han et al. 2022; Kang et al. 2021; Yan et al. 2023; Zhou et al. 2023), although others found no diversity change (Zhang et al. 2023a; Lee et al. 2022; Picca et al. 2019). Thus, the specific makeup of the microbial community may be a more significant factor than its overall diversity (richness) alone. Conflicting results have been reported for the F/B ratio, likely reflecting differences in sex, geography, or diagnostic criteria across cohorts (Han et al. 2022; Yan et al. 2023; Wang et al. 2023). In addition, Wu et al. (2022) proposed the *Prevotella*/*Bacteroides* (P/B) ratio as an alternative marker, which was elevated in sarcopenia (Wu et al. 2022b). Zhou et al. (2023) reported decreased Firmicutes and increased Proteobacteria, indicating a more inflammatory microbial profile (Zhou et al. 2023).

At the genus level, sarcopenia is repeatedly associated with depletion of beneficial SCFA-producing and metabolically supportive taxa, including *Marvinbryantia*, Ruminococcaceae UCG-10, *Akkermansia*, *Lachnospira*, *Roseburia*, *Eubacterium*, *F. prausnitzii*, *Roseburia inulinivorans*, *Alistipes shahii*, *Blautia*, and *Subdoligranulum* (Han et al. 2022; Kang et al. 2021; Ticinesi et al. 2020; Zhou et al. 2023). In contrast, *Lactobacillus*, *Anaerotruncus*, *Butyricimonas*, *Parabacteroides*, *Escherichia–Shigella*, and *Klebsiella* are frequently enriched, while *Desulfovibrio piger*, *Clostridium symbiosum*, and *Hungatella effluvii* appear more prominent in severe cases, collectively supporting a more pro-inflammatory microbial composition (Lee et al. 2022; Kang et al. 2021; Wang et al. 2022; Zhang et al. 2023a). *Prevotella*, however, remains inconsistently associated across cohorts (Wang et al. 2023; Liu et al. 2023; Lee et al. 2022).

Functional analyses parallel these taxonomic shifts, revealing enrichment of LPS biosynthesis together with reduced amino acid and branched-chain amino acid metabolism, altered glycolytic activity, and disturbances in aromatic amino acid and lipid pathways (Kang et al. 2021; Liu et al. 2023; Ticinesi et al. 2020; Wang et al. 2022). In addition, significantly reduced total SCFAs, particularly butyrate and valerate, have been consistently reported and show positive correlations with skeletal muscle mass (Han et al. 2022; Yan et al. 2023). Collectively, these findings highlight impaired microbial butyrogenesis and broader inflammatory metabolic remodeling as recurrent functional features of sarcopenia.

Several predictive models have further highlighted the diagnostic value of these microbial alterations, including classifiers based on the P/B ratio, *Coprococcus*, *Lachnospiraceae*, *D. piger*, *Blautia*, and *Subdoligranulum*, supporting the feasibility of microbiota-based biomarkers for early sarcopenia detection (Wu et al. 2022b; Wang et al. 2022; Zhou et al. 2023).

Frailty, though related to sarcopenia, represents a broader decline involving multiple physiological systems and increased vulnerability to adverse outcomes (Wen et al. 2024; Wang and Wu 2021). Gut microbiota alterations have likewise been repeatedly associated with frailty. Across multiple cohorts, frail older adults show lower microbial diversity together with depletion of *Lactobacillus*, *Bacteroides*/*Prevotella*, *F. prausnitzii*, *Faecalibacterium*, *Prevotella*, *Roseburia*, *P. copri*, and *Coprococcus eutactus*, whereas Enterobacteriaceae, *Eggerthella*, *Coprobacillus*, *Escherichia–Shigella*, *Akkermansia*, *Klebsiella*, *Bacteroides*, *Clostridium hathewayi*, and *Parabacteroides* are repeatedly enriched (van Tongeren et al. 2005; Jackson et al. 2016; Maffei et al. 2017; Xu et al. 2021; Lim et al. 2021; Guo et al. 2022). These microbial patterns are accompanied by elevated IL-6 and zonulin, enrichment of oxidative stress pathways, and evidence of intestinal barrier dysfunction. Although the increased abundance of *A. muciniphila* may reflect excessive mucin degradation under dysbiotic conditions, its disease-context dependence should be interpreted cautiously (Desai et al. 2016).

At the metabolite level, frailty is consistently associated with decreased SCFAs, primary BAs, and tryptophan metabolites, coupled with increased TMAO, SBA, PCS, and methylglyoxal, indicating broad inflammatory, endothelial, and metabolic stress (Ghosh et al. 2020; He et al. 2020).

Sarcopenia and frailty exhibit a partially overlapping gut microbial configuration associated with reduced butyrate availability, impaired amino acid metabolic support, increased endotoxin burden, and metabolite profiles linked to inflammatory and oxidative stress. These recurrent microbial-functional associations parallel diminished muscle maintenance, compromised barrier integrity, and broader physiological vulnerability in older adults. Although individual taxonomic markers vary across cohorts, the consistent overlap in microbial metabolic disruption highlights the gut microbiota as a potential biomarker reservoir for identifying functional decline and resilience loss during aging.

## Comparative microbial signatures and their biomarker potential across age-related diseases

**Fig. 2:**
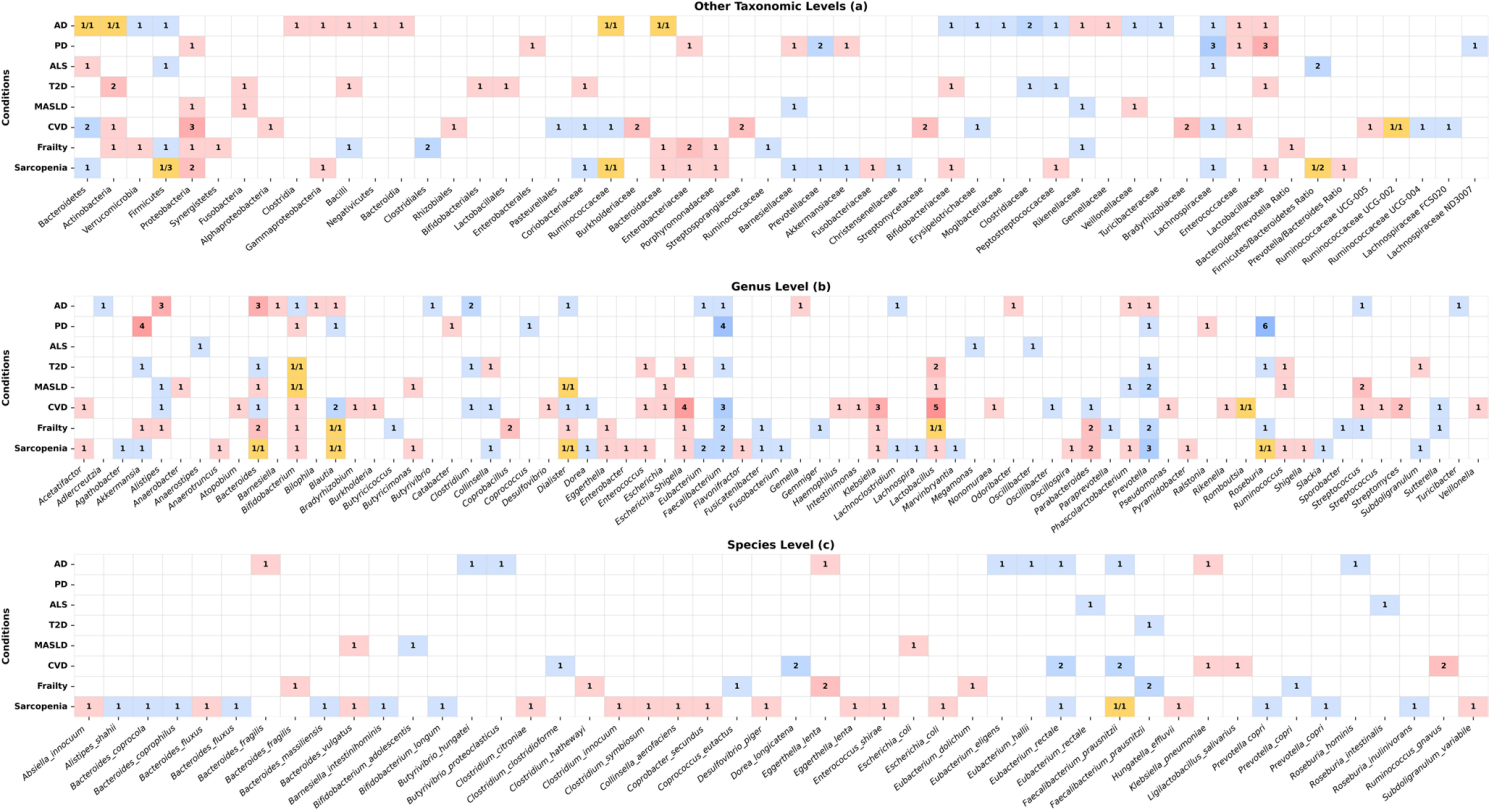
Comparative overviews of microbial taxa associated with age-related diseases and their potential biomarker relevance. Microbial taxa reported as significantly altered across major age-associated disorders (Alzheimer’s disease (AD), Parkinson’s disease (PD), amyotrophic lateral sclerosis (ALS), type 2 diabetes (T2D), metabolic dysfunction-associated steatotic liver disease (MASLD), cardiovascular disease (CVD), frailty, and sarcopenia) are summarized across three taxonomic levels (**a**: higher taxa, **b**: genus, and **c**: species). Color coding denotes direction of change (red, increased; blue, decreased; yellow, conflicting findings). Values within cells indicate the number of independent studies reporting each trend, whereas in yellow cells the fractional format represents the number of studies reporting increased abundance/decreased abundance

Broadly shared microbial patterns are evident across neurodegenerative, metabolic, and musculoskeletal disorders, indicating that many reported alterations reflect a recurrent age-associated dysbiotic core rather than entirely disease-exclusive microbial signatures. This common configuration includes reduced abundance of anti-inflammatory SCFA-producing taxa, enrichment of facultative anaerobes and endotoxin-associated bacteria, and parallel functional shifts toward diminished butyrate and propionate synthesis, increased TMAO and secondary bile acid production, and enhanced oxidative stress-, sulfur metabolism-, and LPS-related pathways. Despite their distinct clinical manifestations, these disorders converge on a similar microbiota-associated background linked to systemic inflammation, metabolic inflexibility, and immune dysregulation. On this basis, microbial taxa repeatedly reported as altered in neurodegenerative (AD, PD, ALS), metabolic (T2D, MASLD, CVD), and musculoskeletal (frailty and sarcopenia) conditions were compiled and directly compared across taxonomic levels in Fig. 2. This comparative synthesis highlights both recurrent cross-disease microbial features and taxa that remain context-dependent or relatively disease-specific.

At higher taxonomic levels, recurrent increases were observed in the families Enterococcaceae, Enterobacteriaceae, and Lactobacillaceae, as well as in the phyla Proteobacteria and Actinobacteria, indicating expansion of facultative or opportunistic taxa commonly associated with oxidative stress and systemic inflammation. In contrast, Clostridiaceae, Lachnospiraceae, Prevotellaceae, and the phylum Firmicutes were repeatedly decreased, reflecting depletion of core anaerobic commensals involved in butyrate production and epithelial homeostasis. Bacteroidetes and Ruminococcaceae exhibited inconsistent or bidirectional trends across studies, suggesting sensitivity to host-related and methodological variation rather than a uniform pathological association.

At the genus level, reproducible increases were observed for *Ruminococcus*, *Lactobacillus*, *Klebsiella*, *Escherichia–Shigella*, and *Enterococcus*, genera frequently associated with mucosal inflammation, endotoxin release, and redox imbalance. Decreases were consistently reported for *Clostridium*, *Faecalibacterium*, *Prevotella*, and *Roseburia*, which are dominant butyrate producers involved in maintaining gut barrier integrity and immune homeostasis. However, these genus-level associations should be interpreted cautiously because substantial heterogeneity exists within several genera; for example, *Ruminococcus* includes both pro-inflammatory species such as *R. gnavus* and beneficial fiber-degrading commensals such as *R. bromii* (Kandasamy et al. 2023; Ze et al. 2012).

Several genera exhibited variable or disease-specific patterns. *Dialister* and *Blautia* showed inconsistent trends across studies. *Bifidobacterium* displayed no uniform direction, with both increases and decreases reported depending on disease context and study design. *Bacteroides* and *Alistipes* demonstrated bidirectional shifts across different disorders. The role of *Akkermansia* appears highly context-dependent, being frequently enriched in PD yet reduced in T2D and sarcopenia.

At the species level, taxa such as *K. pneumoniae*, *E. coli*, *E. lenta*, *B. vulgatus*, and *B. fragilis* were repeatedly enriched in multiple disorders, suggesting a shared association with intestinal inflammation, endotoxin production, and epithelial dysfunction. Conversely, *F. prausnitzii*, *E. rectale*, and *P. copri* were commonly depleted, highlighting recurrent loss of butyrate-producing and anti-inflammatory species linked to healthy gut function.

These comparisons suggest that a subset of recurrently altered taxa forms the core of a recurrent inflammaging-associated dysbiotic profile. This profile is marked by enrichment of facultative and pro-inflammatory taxa, including *Escherichia–Shigella*, *E. lenta*, and *K. pneumoniae*, together with depletion of key butyrate-producing commensals such as *Faecalibacterium*, *E. rectale*, and *Roseburia*. Rather than supporting a single universal microbiome signature for every age-related disorder, the evidence indicates repeated convergence on certain inflammatory and metabolically unfavorable microbial configurations across distinct disease settings.

At the same time, the heterogeneity observed across studies underscores the substantial influence of population structure, dietary background, medication exposure, and methodological differences. Recent perspectives similarly emphasize that many microbiome–disease associations may be consequential rather than directly causative, as recurrent depletion of commensals and enrichment of pathobionts are observed across multiple chronic disorders, suggesting shared dysbiotic tendencies rather than entirely disease-specific patterns (O’Toole 2024). Therefore, while these recurrent taxa provide a useful comparative reference framework, standardized age-stratified and longitudinal analyses remain necessary to separate shared aging-associated dysbiosis from truly disease-enriched microbial alterations.

## Methodological approaches in microbiota biomarker discovery

**Fig. 3:**
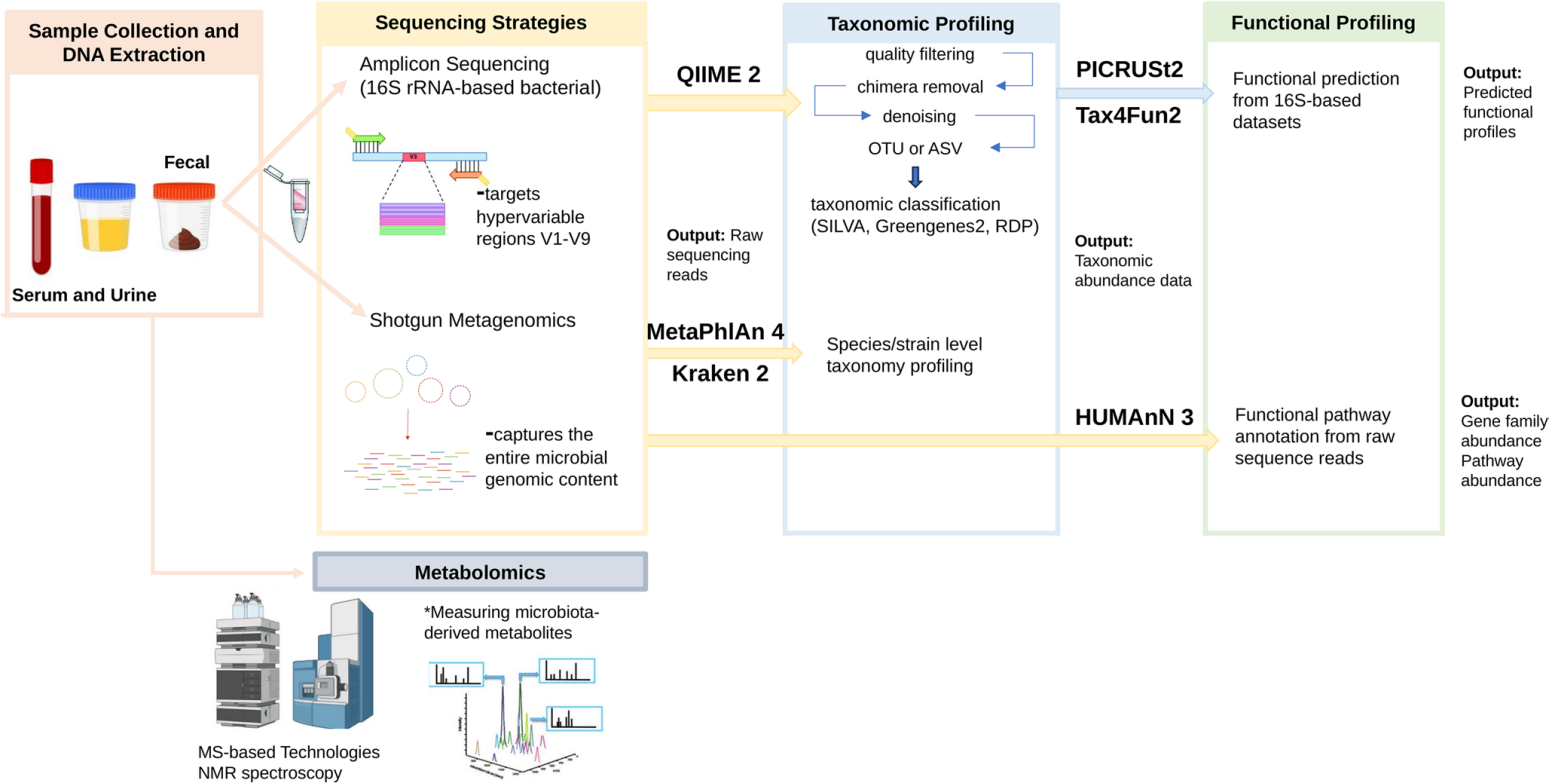
Overview of a bacterial microbiota-focused multi-omics workflow for gut microbial biomarker discovery. Sequential steps include sample collection, sequencing (16S rRNA amplicon or shotgun metagenomics), taxonomic and functional profiling, and metabolomic integration. Representative tools are indicated for each stage

Advances in next-generation sequencing technologies have greatly improved the identification of microbiome-derived biomarkers by enabling comprehensive characterization of taxonomic and functional changes associated with aging and age-related diseases. The discovery of such biomarkers relies on a systematic workflow that integrates sample collection, sequencing-based community profiling, bioinformatic processing, functional inference, and metabolite detection (Fig. 3). Each of these methodological layers contributes to the accuracy, reproducibility, and interpretability of biomarker discovery.

Fecal material remains the most commonly used matrix in human microbiota studies due to its non-invasive collection and ability to represent luminal microbial communities (Fu et al. 2016). However, it does not fully capture mucosa-associated taxa that can be accessed through targeted intestinal biopsies in specific clinical contexts. Variations in sampling, preservation, and DNA extraction procedures are critical sources of technical bias, potentially affecting both taxonomic and functional readouts (Levitan et al. 2023). Therefore, standardization of these steps is essential for reliable biomarker comparison across studies and cohorts.

Two complementary sequencing approaches are widely employed to characterize microbial communities: amplicon-based and shotgun metagenomic sequencing. Amplicon sequencing targets hypervariable regions of the 16S rRNA gene, providing taxonomic information at the genus or sometimes species level, whereas shotgun metagenomics captures the entire microbial genomic content without prior amplification (Calle 2019). The latter provides higher taxonomic resolution and, importantly, direct access to functional gene repertoires. The choice between these strategies depends on the required resolution, sequencing depth, and study design, as each approach involves trade-offs between coverage, cost, and interpretability.

Following sequencing, bioinformatic pipelines transform raw reads into structured taxonomic profiles. For amplicon data, this process is commonly implemented using platforms such as QIIME 2 (Bolyen et al. 2018), which include quality filtering, chimera removal, and denoising to generate ASVs or OTUs, followed by taxonomic classification against curated databases such as SILVA (Pruesse et al. 2007) or the more recently updated Greengenes2 (McDonald et al. 2024), while legacy databases such as Greengenes (DeSantis et al. 2006) and RDP (Maidak et al. 2000) are now less frequently used. Shotgun datasets, in contrast, rely on genome-based classifiers such as MetaPhlAn 4 (Blanco-Míguez et al. 2023) and Kraken 2 (Wood et al. 2019), which assign taxonomy based on unique marker genes or k-mer signatures. The accuracy and consistency of these reference databases directly influence the comparability of microbial signatures across studies, underscoring the need for updated and standardized reference resources.

Beyond taxonomic profiling, the functional dimension of the microbiota provides deeper insight into its interaction with the host. In 16S-based datasets, functional inference can be achieved using predictive algorithms such as PICRUSt2 (Douglas et al. 2020) or Tax4Fun2 (Wemheuer et al. 2020), which extrapolate putative gene content from phylogenetic similarity to reference genomes. Although these predictions are informative, they remain limited by the completeness of available annotations. Shotgun metagenomics, on the other hand, enables direct functional profiling through annotation of sequencing reads or assembled contigs against databases such as KEGG (Kanehisa and Goto 2000), eggNOG (Huerta-Cepas et al. 2019), or MetaCyc (Caspi et al. 2020), typically implemented using pipelines such as HUMAnN 3 (Beghini et al. 2021). This approach yields high-resolution information on microbial metabolic pathways, including those related to SCFA synthesis, BA conversion, and amino acid metabolism, all processes relevant to host aging biology.

Complementary to sequencing-based methods, metabolomics provides biochemical evidence of host–microbiome interactions. Microbiota-derived metabolites can be measured in feces, serum, or urine using targeted or untargeted approaches based on mass spectrometry-based metabolomics (Luan et al. 2019) such as LC–MS/MS (Sun et al. 2023), GC–MS/MS (Denisov et al. 2025), or NMR spectroscopy (Zacharias et al. 2022). Targeted analyses quantify specific metabolites such as SCFAs, BAs, or tryptophan derivatives, whereas untargeted profiling captures the broader metabolome (Bingol 2018). Integrating metabolomic readouts with genomic and functional data enables more precise identification of biomarkers that reflect both microbial potential and realized metabolic activity.

The integration of taxonomic, functional, and metabolomic information within a unified analytical framework provides a powerful means of discovering microbiome-based biomarkers. This multi-omics approach facilitates movement from descriptive community profiling toward mechanistic and clinically relevant interpretations of the microbiota’s role in aging. By linking microbial structure to function and to measurable host outcomes, such integrative analyses offer a foundation for developing reliable microbial and metabolite-based indicators of biological aging.

### Bioinformatics and machine learning for age-associated biomarker modeling

The complexity and high dimensionality of microbiome data require advanced computational strategies for the identification and validation of age-related biomarkers. Bioinformatics provides the foundation for these analyses by enabling the preprocessing, integration, and interpretation of large-scale sequencing datasets. Raw reads generated from amplicon or shotgun metagenomic sequencing are subjected to standardized pipelines for quality control, host read removal, taxonomic assignment, and functional annotation. The resulting multidimensional datasets (microbial taxa, gene functions, and metabolic pathways) serve as input for statistical (Calle 2019) and machine learning models (Li et al. 2022) designed to uncover microbial signatures associated with chronological or biological aging.

Because microbiome data are compositional and sparse, normalization and transformation are critical steps prior to statistical modeling. Approaches such as cumulative sum scaling, centered log-ratio transformation, or variance-stabilizing normalization are commonly applied to reduce biases related to sequencing depth (Gloor et al. 2017; Weiss et al. 2017). Exploratory analyses using ordination methods (e.g., principal component analysis (PCA), non-metric multidimensional scaling (NMDS), canonical correspondence analysis (CCA) help visualize global community differences across age groups (Ramette 2007). Both alpha diversity metrics (e.g., Shannon, Chao1, Simpson) and beta diversity measures (e.g., Bray–Curtis dissimilarity, UniFrac distance) provide complementary insights into ecosystem structure and resilience, reflecting within-sample richness and between-sample compositional variation, respectively (Lozupone et al. 2012; Calle 2019).

Feature selection and modeling aim to identify microbial taxa, genes, or pathways most strongly associated with aging (Papoutsoglou et al. 2023). Differential abundance testing is widely used in microbiome research, including aging-related studies, to identify taxa or functional pathways associated with chronological or biological age. While DESeq2 (Love et al. 2014) has been historically used, it is now generally discouraged due to limitations related to sparsity and compositionality, whereas compositionally aware approaches such as ALDEx2 and ANCOM-II are preferred (Nearing et al. 2022). These selected features are then incorporated into supervised learning algorithms, including random forests (RF) (Huang et al. 2020), support vector machines (SVM) (Lan et al. 2013), or gradient boosting (XGB) (Shen and Zhang 2022), to classify samples by age group or predict biological age (Chen et al. 2022). Deep learning approaches have recently advanced microbiome-based biomarker discovery by capturing complex, non-linear patterns in high-dimensional data. Models such as DeepMicro use autoencoder-based architectures to derive denoised representations of microbial communities, improving phenotype classification and inspiring age-prediction frameworks (Oh and Zhang 2020). Hybrid architectures like phyLoSTM (Sharma and Xu 2021), combining convolutional neural networks (CNNs) and long short-term memory (LSTM) layers, leverage both taxonomic and temporal structure, while MDeep (Wang et al. 2021a) integrates phylogenetic tree depth through hierarchical convolutional layers to encode microbial relationships more effectively. These designs indicate that incorporating biological context (such as taxonomic proximity or temporal dependency) enhances model interpretability and predictive performance.

Specialized microbiome aging clocks now employ these architectures as regressors for host age. The deep neural network model by Galkin et al. (2020), trained on over 4,000 human gut metagenomes (ages 18–90), achieved a mean absolute error of ~ 5.9 years (Galkin et al. 2020). Subsequent frameworks such as gAge and other multi-view ensemble models combined taxonomic and functional features to improve prediction accuracy and revealed associations between microbiome-predicted age residuals, frailty, and health status (Wang et al. 2024). These results suggest that microbial configurations can approximate biological age and reflect physiological aging.

Emerging transformer-based architectures further extend this field. Their attention mechanisms enable modeling of long-range interactions among hundreds of taxa, producing context-aware embeddings with improved performance over traditional models. Early implementations like MetaTransformer have successfully integrated metagenomic and metabolomic layers, illustrating the potential of transformer-driven frameworks to refine microbiome-based aging clocks (Przymus et al. 2025). Model robustness depends on rigorous validation (Nearing et al. 2022). Cross-validation within datasets is essential, but true generalizability requires independent external cohorts analyzed through harmonized pipelines. Addressing batch effects, population heterogeneity, and metadata inconsistency remains a major challenge for reproducible biomarker development.

In summary, bioinformatics and machine learning approaches have substantially advanced microbiome research from descriptive community profiling into predictive modeling. When coupled with network and multi-omics analyses, these computational frameworks provide an effective framework for identifying microbial signatures linked to biological aging and advancing the development of reliable, data-driven biomarkers. Future biomarker modeling frameworks will increasingly rely on standardized pipelines, interoperable datasets, and transparent model benchmarking to ensure reproducibility and clinical translation.

## Conclusion and future perspectives

Current evidence consistently indicates that gut microbiota composition and function show substantial differences across healthy, pathological, and advanced-age cohorts, reflecting both physiological adaptation and pathological decline. Recurrent microbial signatures, including reduced abundance of SCFA producers and increased prevalence of pro-inflammatory taxa, are consistently associated with frailty, inflammaging, and age-related diseases. In contrast, individuals exhibiting healthy or exceptional longevity often maintain metabolically flexible and anti-inflammatory microbial networks. These findings support the view that gut microbiota profiles and derived metabolites represent candidate biomarkers of biological aging.

Nevertheless, most existing studies remain descriptive and cross-sectional, limiting the ability to infer causality. The predictive value of microbial and metabolite-based biomarkers requires validation in longitudinal cohorts using standardized sampling and analytical frameworks. Integration of taxonomic, functional, and metabolomic layers through multi-omics approaches will be crucial for identifying robust and reproducible biomarker panels. Equally important will be the establishment of reference datasets across populations to distinguish universal from population-specific microbial aging signatures. At the same time, the substantial heterogeneity observed across current cohorts indicates that standardization and age-stratified validation remain prerequisites before any universal microbiome aging biomarker can be reliably defined.

Future research should prioritize translation of microbiome-derived biomarkers into clinically applicable tools for assessing biological age, monitoring intervention outcomes, and stratifying disease risk, while also expanding beyond bacterial components to include domains such as the gut virome and mycobiome. This objective will require harmonized bioinformatic pipelines, rigorous statistical validation, and integration with established molecular and physiological markers of aging. Through such coordinated efforts, microbiota-based biomarkers may contribute to more precise and individualized models of healthy and pathological aging while supporting the development of biotechnology-driven interventions, including rationally designed microbial consortia, next-generation probiotics and live biotherapeutic products, and precision fecal microbiota transplantation strategies.

## Data Availability

No datasets were generated or analysed during the current study.
